# Neonatal blood stream infections in tertiary referral hospitals in Kurdistan, Iran

**DOI:** 10.1186/s13052-015-0136-4

**Published:** 2015-06-09

**Authors:** Bahram Nikkhoo, Fariba Lahurpur, Ali Delpisheh, Mohammad Aziz Rasouli, Abdorrahim Afkhamzadeh

**Affiliations:** Department of Pathology, Medical Faculty, Kurdistan University of Medical Sciences, Sanandaj, Iran; Department of Microbiology, Kurdistan University of Medical Sciences, Sanandaj, Iran; Department of Clinical Epidemiology, Ilam University of Medical Sciences, Ilam, Iran; Department of Epidemiology & Statistics, Kurdistan University of Medical Sciences, Sanandaj, Iran; Department of Community Medicine & Kurdistan Research Center for Social Determinants of Health, Kurdistan University of Medical Sciences, Sanandaj, Iran

**Keywords:** Bloodstream Infections (BSI), Neonatal Intensive Care Unit (NICU), Sanandaj

## Abstract

**Background:**

Bloodstream infection (BSI) is one of the most common causes of nosocomial infection in neonatal intensive care units (NICU). The aim of the present study was to determine bacterial agents and their susceptibility patterns to antibiotics and to investigate the risk factors associated with BSI.

**Methods:**

This was a nested case–control study carried out from September 2009 to June 2010 in the NICU wards in Sanandaj hospitals western Iran. Cases were patients with BSI and controls were other patients who had negative blood culture. Bacteriologic diagnosis and antibiotic susceptibility pattern was performed based on the Edward & Ewings and the National Committee of Clinical Laboratory (NCCL) Standards.

**Results:**

Of 472 patients who hospitalized in NICU, 6.4% had BSI (n = 30) including 17girls (56.7%) and 13 boys (43.3%). Enterobacter *SPP* was the predominant isolated bacteria from blood culture (36.7%). The maximum antibiotic resistance and sensitivity were observed by Tetracycline and Ciprofloxacin respectively. Risk factors associated with BSI were age ≤ 7 days (p = 0.001), previous antibiotic consumption (p = 0.013), and low birth weight (LBW), (p = 0.001).

**Conclusions:**

Gram negative bacteria and Entrobacter in particular are the most common pathogens. Improving prenatal health care, standards of infection control and choosing accurate antibiotics are recommended to avoid BSI in neonatal intensive care units.

## Introduction

The World Health Organization has estimated that 130 million neonates are born each year and more than 10 millions die during the first five days after birth. Bloodstream infection is one of the major causes of mortality in developing countries and in some communities almost half of patients in intensive care units acquire infection [1,2]. Hospital infections especially in the neonatal wards are the major cause of mortality in children [3,4].

Neonatal infection can be acquired vertically from birth canal bacteria or environmentally due to lack health facilities. Neonatal septicemia is a clinical syndrome bacteremia with symptoms and clinical signs in the first months of life and delay in its diagnosis and treatment causes mortality [5]. Signs and symptoms of neonatal infection are often nonspecific, and the rates of hospital septicemia operating with both prematurity and low birth weight are increasing [6-8].

Overall, 16.5% of all births in the world are growth restricted annually [9]. It is estimated that one in five neonates in developing countries is suffering from septicemia and blood infection [10]. The organism spectrum of neonatal septicemia can change over time and is different from region to region. Septicemia with gram-negative microorganisms is expanding especially in Asia, but it is still the main cause of septicemia in developing countries [11,12].

Newborns are devoiced of efficient structural barriers, of a protective endogenous microbial flora and of a mature immune system. Premature and growth restricted neonates in particular who admitted to NICUs are under a profound physiologic instability and are frequently exposed to therapeutic interventions associated with infectious complications such as invasive procedures and broad-spectrum antibiotics [13].

The organisms responsible for neonatal sepsis vary across geographical boundaries and in time of onset. One organism alone or a group of organisms may replace over time as the leading cause of neonatal sepsis in a particular region. It is crucial to monitor the local epidemiology of neonatal BSI continuously to detect any changes in patterns of infection and susceptibility to various antibiotics.

The present study was designed and implemented to determine the etiology of BSI and to examine antibiotic susceptibility patterns of the isolated organisms as well as risk factors for septicemia.

## Methods

The nested case–control study was conducted in the NICU of general teaching hospitals and tertiary care referral centers in Sanandaj city, Kurdistan province, western Iran. All neonates admitted to the NICU from September 2009 to June 2010 were recruited. Cases were patients with blood stream infection (BSI) and controls were patients with negative blood cultures. Bloodstream infections were confirmed when at least a positive peripheral-blood culture was present.

The antibiotic susceptibility for isolated pathogens was determined and met all the recommendations of the National Committee of Clinical Laboratory Standards breakpoint values [14]. Bacteriologic diagnosis and antibiotic susceptibility pattern was performed based on Edward & Ewings [15].

Clinical signs of sepsis were the following: temperature >38°C or <36°C, heart rate >20/min or inflammatory response syndrome (SIRS). Low birth weight was defined as weight of ≤ 2000 grams.

Factors associated with blood infections, including demographic characteristics such as age, sex, occupation and education of parents and other factors such as hospitalization period, previous antibiotic consumption, low birth weight and the using ventilator were investigated.

Chi-square test was performed to assess potential risk factors and neonatal bloodstream. The level of statistical significance adopted was p < 0.05. SPSS version17 was used for all statistical analysis.

## Results

**Figure 1:**
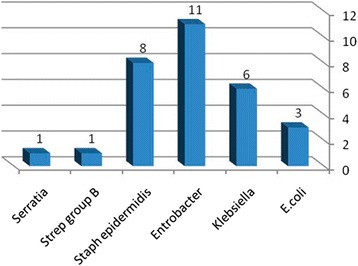
Frequency of isolated bacteria in Neonates hospitalized in NICU.

Of 472 patients recruited 30 BSI cases were diagnosed. The total BSI incidence rate was 6.4%. Mean age and standard deviation was 12.13 ± 5.72 in BSI group and 14.57 ± 6.03 in non BSI group (p = 0.03) . In BSI group, 17 (56.7%) were girls and 13 (43.3%) were boys with no significant difference (p > 0.05). The isolated bacteria identified in blood culture of 30 neonates are showen in (Figure 1) including Enterobacter (36.7%), Klebsiella (20.0%), Escherichia coli (10.0%) and Staphylococcus epidermidis (26.7%) and others (6.6%) .

Susceptibility testing results are showen in Table 1. Klebsiella species revealed high susceptibility to ciprofloxacin (98%), cotrimoxazole (60%). Enterobacter isolates were susceptible to cotrimoxazole (90%) and ciprofloxacin (88.9%) and amikacin (71.4, but less so to the cefalotin/ampicillin. E. coli revealed decreased susceptibility to amikacin (100%), cefotaxime/cefalotin (66.7%), cefteriaxone/cefixim/cotrimoxazole/gentamicin (33.3%), tetracycline (0%).

**Table 1:** **Results of susceptibility for isolated bacteria (N = 30)**

**Bacteria**	**E.coli**	**Klebceilla**	**Entrobacter**	**Staph Epidermidis**	**Strep GB**	**Serratia**
**Antibiotic**	**N (%)**	**N (%)**	**N (%)**	**N (%)**	**N (%)**	**N (%)**
	**Susceptible**	**Susceptible**	**Susceptible**	**Susceptible**	**Susceptible**	**Susceptible**
**Ciprofloxacin**	1 (50)	5 (100)	8 (88.9)	4 (80)	*	1 (100)
**Cefotaxime**	2 (66.7)	0 (0)	6 (66.7)	3 (60)	0 (0)	1 (100)
**Cefalotin**	2 (66.7)	0 (0)	1 (12.5)	1 (25)	*	1 (100)
**Ampicillin**	1 (33.3)	0 (0)	1 (11.1)	0 (0)	1 (100)	0 (0)
**Amikacin**	3 (100)	0 (0)	5 (71.4)	*	*	1 (100)
**Cefixim**	1 (33.3)	0 (0)	6 (66.7)	2 (40)	0 (0)	1 (100)
**Cefteriaxone**	1 (33.3)	3 (60)	5 (50)	4 (80)	0 (0)	1 (100)
**Cotrimoxazole**	1 (33.3)	0 (0)	9 (90)	2 (66.7)	0 (0)	1 (100)
**Getamicin**	1 (33.3)	0 (0)	4 (57.1)	0 (0)	*	1 (100)
**Tetracycline**	0 (0)	0 (0)	4 (44.4)	0 (0)	0 (0)	0 (0)

*Not tested for susceptibility to this antibiotic.

The variables associated with BSI according to the univariate analysis were:

age ≤ 7 days (p = 0.001), previous antibiotic consumption (p = 0.013), and birth weight ≤ 2 kg (LBW), (p = 0.001).

On the other hand, sex, ventilator use, clinical signs and existence of underlying disease were not significantly associated with BSI (Table 2).

**Table 2:** **Demographic and clinical characteristics of neonates**

**Variable**	BSI**	**Non-BSI**	**P Value**	Odds Ratio (CI***)
	**N (%)**	**N (%)**		
**Birth weight**			0.001	4.55 (1.95-10.64)
≥**2000 g**	21 (4.9)	404 (95.1)		
**<2000 g**	9 (19.1)	38 (80.9)		
**Ventilator**			0.49*	
**NO**	8 (7.8)	95 (92.2)		
**YES**	22 (6)	347 (94)		
**Previous antibiotic use**			0.01	2.65 (1.21-5.79)
**NO**	10 (3.8)	252 (96.2)		
**YES**	20 (9.5)	190 (90.5)		
**Clinical Sign of Sepsis**			0.49*	
**NO**	1 (2.6)	38 (97.4)		
**YES**	29 (6.7)	404 (93.3)		
**Underlying disease**			0.7*	
**NO**				
**YES**	29 (6.6)	409 (93.4)		
	1 (2.9)	33 (97.1)		
**Age group**			0.001	0.22 (0.1-0.5)
≤**7 days**	11(17.7)	51 (82.3)		
**>7 days**	19 (4.6)	391 (95.4)		

*Not significance.

**Blood stream infection.

*** Cl, confidence interval.

## Discussion

Blood infection is the main cause of neonatal mortality in the neonatal intensive care units [16]. The prevalence rate of blood stream infection in the present study was 6.4%. The majority of pathogens isolated from blood cultures were Gram-negative bacteria (70%) including Enterobacter, Klebsiella and Escherichia coli. Risk factors associated with sepsis were age ≤ 7 days, previous antibiotic consumption, and low birth weight.

Prevalence of neonatal septicemia, its pathogens and risk factors are different across the world. Macharashvili and colleagues has reported a prevalence rate of 63% for septicemia which Gram-negative bacteria was the highest [2]. Similar studies in Iran [3,17] and other countries [18-20] are consistent with the result of our study except that the prevalence of septicemia in the present study was much lower. Because our patients were whom hospitalized in NICU for any reason, but in other studies all of patients had clinical signs for sepsis.

The significant relationship between lengths of hospitalization more than a week and blood infection in the present study, was also consistent with the recent reports [7,8].

In the present study, Klebsiella species revealed high susceptibility to ciprofloxacin (98%). Enterobacter isolates were susceptible to cotrimoxazole and ciprofloxacin. E. coli revealed gighest susceptibility to amikacin and the least to tetracycline (0%).

In a Palestinian study in Gaza, the most sensitive antibiotics were Meropenem and isolates were resistant against other antibiotics including Ciprofloxacin [5]. The pattern of sensitivity to gram-negative bacteria BSI in the present study was also differs from the recent report by Macharashvili for Ciprofloxacin, Carbapnem and Gentamicin [2]. In another study, Staphylococcus aureus and Escherichia coli have been reported as the most common cause of blood infection [21].

In this study, age ≤ 7 days (p = 0.001), previous antibiotic consumption (p = 0.013), and low birth weight, (p = 0.001) were significantly associated with BSI. Similar findings have been reported in previous studies [4,8,10].

We had one limitation: Information on gestational age was not available.

## Conclusions

Gram negative bacteria and Entrobacter in particular are the most common pathogens. Improving prenatal health care, standards of infection control and choosing accurate antibiotics are recommended to avoid BSI in neonatal intensive care units.

## Abbreviation

BSI: Bloodstream infection
NICU: neonatal intensive care units
